# Association of *MAPT* haplotypes with Alzheimer’s disease risk and *MAPT* brain gene expression levels

**DOI:** 10.1186/alzrt268

**Published:** 2014-07-01

**Authors:** Mariet Allen, Michaela Kachadoorian, Zachary Quicksall, Fanggeng Zou, High Seng Chai, Curtis Younkin, Julia E Crook, V Shane Pankratz, Minerva M Carrasquillo, Siddharth Krishnan, Thuy Nguyen, Li Ma, Kimberly Malphrus, Sarah Lincoln, Gina Bisceglio, Christopher P Kolbert, Jin Jen, Shubhabrata Mukherjee, John K Kauwe, Paul K Crane, Jonathan L Haines, Richard Mayeux, Margaret A Pericak-Vance, Lindsay A Farrer, Gerard D Schellenberg, Joseph E Parisi, Ronald C Petersen, Neill R Graff-Radford, Dennis W Dickson, Steven G Younkin, Nilüfer Ertekin-Taner

**Affiliations:** 1 Department of Neuroscience, Mayo Clinic Florida, Jacksonville, FL 32224, USA; 2 Department of Health Sciences Research, Mayo Clinic Minnesota, Rochester, MN 55905, USA; 3 Department of Health Sciences Research, Mayo Clinic Florida, Jacksonville, FL 32224, USA; 4 Medical Genome Facility, Mayo Clinic Minnesota, Rochester, MN 55905, USA; 5 Department of Medicine, University of Washington, Seattle 98104, WA, USA; 6 Departments of Biology, Neuroscience, Brigham Young University, Provo, UT 84602, USA; 7 Department of Molecular Physiology and Biophysics, and the Vanderbilt Center for Human Genetics Research, Vanderbilt University, Nashville, TN, USA; 8 Department of Epidemiology and Biostatistics, Case Western Reserve University, Cleveland, OH 44106, USA; 9 Gertrude H. Sergievsky Center, Department of Neurology, and Taub Institute on Alzheimer’s Disease and the Aging Brain, Columbia University, New York, NY, USA; 10 The John P. Hussman Institute for Human Genomics and Dr. John T. Macdonald Foundation Department of Human Genetics, University of Miami, Miami, FL, USA; 11 Departments of Biostatistics, Medicine (Genetics Program), Ophthalmology, Neurology, and Epidemiology, Boston University, Boston, MA, USA; 12 Department of Pathology and Laboratory Medicine, University of Pennsylvania Perelman School of Medicine, Philadelphia, PA, USA; 13 Department of Laboratory Medicine and Pathology, Mayo Clinic, Rochester, MN 55905, USA; 14 Department of Neurology, Mayo Clinic Minnesota, Rochester, MN 55905, USA; 15 Department of Neurology, Mayo Clinic Florida, 4500 San Pablo Road, Birdsall 3, Jacksonville, FL 32224, USA

## Abstract

**Introduction:**

*MAPT* encodes for tau, the predominant component of neurofibrillary tangles that are neuropathological hallmarks of Alzheimer’s disease (AD). Genetic association of *MAPT* variants with late-onset AD (LOAD) risk has been inconsistent, although insufficient power and incomplete assessment of *MAPT* haplotypes may account for this.

**Methods:**

We examined the association of *MAPT* haplotypes with LOAD risk in more than 20,000 subjects (*n*-cases = 9,814, *n*-controls = 11,550) from Mayo Clinic (*n*-cases = 2,052, *n*-controls = 3,406) and the Alzheimer’s Disease Genetics Consortium (ADGC, *n*-cases = 7,762, *n*-controls = 8,144). We also assessed associations with brain *MAPT* gene expression levels measured in the cerebellum (*n* = 197) and temporal cortex (*n* = 202) of LOAD subjects. Six single nucleotide polymorphisms (SNPs) which tag *MAPT* haplotypes with frequencies greater than 1% were evaluated.

**Results:**

H2-haplotype tagging rs8070723-G allele associated with reduced risk of LOAD (odds ratio, OR = 0.90, 95% confidence interval, CI = 0.85-0.95, p = 5.2E-05) with consistent results in the Mayo (OR = 0.81, p = 7.0E-04) and ADGC (OR = 0.89, p = 1.26E-04) cohorts. rs3785883-A allele was also nominally significantly associated with LOAD risk (OR = 1.06, 95% CI = 1.01-1.13, p = 0.034). Haplotype analysis revealed significant global association with LOAD risk in the combined cohort (p = 0.033), with significant association of the H2 haplotype with reduced risk of LOAD as expected (p = 1.53E-04) and suggestive association with additional haplotypes. *MAPT* SNPs and haplotypes also associated with brain *MAPT* levels in the cerebellum and temporal cortex of AD subjects with the strongest associations observed for the H2 haplotype and reduced brain *MAPT* levels (β = -0.16 to -0.20, p = 1.0E-03 to 3.0E-03).

**Conclusions:**

These results confirm the previously reported *MAPT* H2 associations with LOAD risk in two large series, that this haplotype has the strongest effect on brain *MAPT* expression amongst those tested and identify additional haplotypes with suggestive associations, which require replication in independent series. These biologically congruent results provide compelling evidence to screen the *MAPT* region for regulatory variants which confer LOAD risk by influencing its brain gene expression.

## Introduction

Alzheimer’s disease (AD), the most prevalent cause of dementia, is defined by two neuropathological hallmarks: senile plaques primarily composed of extracellular amyloid-beta (Aβ) deposits and intracellular neurofibrillary tangles (NFTs) comprised of hyper-phosphorylated tau protein. *MAPT* (micro-tubule associated protein) encodes tau and resides within a ~900 kilobase (kb) inversion polymorphism (reviewed [1]) that generates a ~1.3 megabase (Mb) region of linkage disequilibrium (LD) defined by two extended haplotypes, referred to as H1 and H2. Variants have evolved that occur on only the H1 haplotype resulting in multiple sub-haplotypes.

Both common and rare genetic variation in *MAPT* have been strongly implicated in primary tauopathies. Rare missense and exon 10 splicing mutations, which lead to increased levels of tau isoforms with four microtubule binding domains (aka 4-repeat or 4R tau) lead to familial frontotemporal dementia with parkinsonism linked to chromosome 17 (FTDP-17) [2,3], whereas the common *MAPT* H1 haplotype strongly associates with increased risk of progressive supranuclear palsy (PSP) and corticobasal degeneration (CBD) [4-8]. A recent genome-wide association study (GWAS) of PSP risk identified *MAPT* as the strongest locus, with risk alleles at rs8070723 which tags the H1 haplotype and also for rs242557, which partially tags the H1c subhaplotype [8].

Despite having tauopathy as a defining lesion, reports of association between AD and genetic variants at the *MAPT* locus are inconsistent. While *MAPT* H1 [9] haplotype or H1c subhaplotype [10-13] showed association with AD risk in some studies, others failed to detect association with H1 [10,13,14], H1c [15] or other *MAPT* variants [16]. The sample size for most of these studies range from a few hundred to a few thousand; and the largest published study of ~17,000 subjects only evaluated the H1/H2 haplotypes but none of the H1-subhaplotypes [9].

In addition to investigations of *MAPT* variants with risk for tauopathies, some studies also assessed their role in gene expression. *MAPT* exons 2, 3, 4a, 6 8 and 10 are known to be alternatively spliced [1], there are FTDP-17 splicing mutations which increase 4R tau [2,3] and 4R tau is increased in affected brain regions in PSP and CBD [17,18]. Allele-specific gene expression studies in human brains and neuronal cell lines identified higher levels of exon 10 containing transcripts but not total *MAPT* associated with the H1-haplotype [19] and higher levels of exon 2- and 3-containing transcripts associated with the H2 haplotype [20]. *MAPT* H1c-subhaplotype was associated with higher total and 4R *MAPT* levels in human brains [11]. A study of exon levels in multiple brain regions from humans identified higher expression levels of exon 3 associated with the H2 haplotype, but no association of *MAPT* levels with the H1c-subhaplotype [21]. We have previously reported association of *MAPT* H1-tagging and rs242557 SNPs with increased brain *MAPT* levels in ~400 brains from a combined cohort of subjects with AD and other brain pathologies [22]. Collectively, these findings suggest that the disease risk conferred by some *MAPT* variants could be due to higher total or 4R tau levels and/or that the protective effect of *MAPT* H2-haplotype might be secondary to an increase in N-terminal exon-containing *MAPT* transcripts. While these studies are informative, to date, there has not been a systematic and well-powered analysis of *MAPT* subhaplotypes for association with *MAPT* brain expression levels.

Herein, we present a comprehensive assessment of *MAPT* variants that tag all *MAPT* subhaplotypes of frequency >1% in the largest to date *MAPT* association study of 9,814 LOAD cases vs. 11,550 controls. Further, we evaluate association of these *MAPT* variants in two brain regions: the cerebellum, which is predominantly unaffected in AD and the typically affected temporal cortex from ~200 autopsied LOAD subjects. Our well-powered and complementary investigation of disease risk and gene expression provides compelling evidence for a role of transcriptional regulatory variants of *MAPT* in conferring LOAD risk.

## Methods

### Subjects and samples

#### Mayo clinic cohort

We evaluated LOAD risk association with *MAPT* variants in 2,052 LOAD cases vs. 3,406 controls from Mayo Clinic. These elderly European-American subjects were from two clinical case–control series recruited at the Mayo Clinic in Rochester, MN (RS series: 615 LOAD cases, 2,425 controls) and Jacksonville, FL (JS series: 886 LOAD cases, 981 controls), as well as 551 autopsy-confirmed LOAD subjects from the Brain Bank at Mayo Clinic Florida (Additional file 1: Table S1). All clinical subjects were evaluated by a Mayo Clinic neurologist and autopsied subjects were diagnosed by our neuropathologist (DWD). All clinical LOAD cases had probable or possible AD and all pathologic LOAD cases had definite AD according to NINCDS-ADRDA criteria [23]. All controls had a clinical dementia rating score of 0. All LOAD subjects had an age at disease diagnosis (clinical), death (autopsied) and controls at their most recent visit ≥60 years. A subset of the Mayo Clinic cohort was included in the Mayo LOAD GWAS [24] (Additional file 1: Table S1) and gene expression GWAS (eGWAS) [22] from the temporal cortex (TCX, n = 202) and cerebellum (CER, n = 197). This study was approved by the Mayo Clinic institutional review board and appropriate informed consent was obtained from all individuals.

#### ADGC cohort

We utilized genetic data and covariate information on the European-American subjects from the Alzheimer’s Disease Genetics Consortium (ADGC) cohort. These subjects were collected from multiple research centers and designated into the following 14 series: Adult Changes in Thought (ACT)/Electronic Medical Records and Genetics (eMERGE), National Institute on Aging (NIA) Alzheimer Disease Centers (ADCs), Alzheimer Disease Neuroimaging Initiative (ADNI), Multi-Site Collaborative Study for Genotype-Phenotype Associations in Alzheimer’s Disease (GenADA), University of Miami/Vanderbilt University/Mt. Sinai School of Medicine (UM/VU/MSSM), MIRAGE Study, Oregon Health and Science University (OHSU), NIA-LOAD, Translational Genomics Research Institute series 2 (TGEN2), Rush University Religious Orders Study/Memory and Aging Project (ROSMAP), University of Pittsburgh (UP), and Washington University (WU). Detailed descriptions of these cohorts are provided elsewhere [25,26].

The ADGC cohort included subjects from the Mayo Clinic. To avoid any overlap, all subjects from Mayo Clinic were removed from the ADGC cohort. Standard quality control (QC) measures were applied to the ADGC dataset [27] with the following cutoffs, 95% call rate per person, 1% minor allele frequency (MAF) and 95% call rate for SNP, Hardy-Weinberg equilibrium (HWE) p > 1E-06 in controls. Additionally, directly observed (not imputed) SNPs from subjects across all series were evaluated for relatedness by using KING (Kinship-based INference for Gwas)-Robust [27] and a single representative was chosen for each pair of individuals who were third degree relatives or closer. Similarly, one representative was chosen for each family for the MIRAGE and NIA-LOAD family based studies. All cohort genotypes were imputed to a common set of >2 million SNPs (HapMap2) by the ADGC, as described [26]. The 7,762 LOAD cases and 8,144 controls from the ADGC (Additional file 1: Table S1), which remained after the QC, were utilized for the *MAPT* variant associations.

### RNA isolation and gene expression measurements

All samples utilized in the brain gene expression analyses in this study are a subset of the previously published Mayo Clinic expression GWAS (eGWAS) [22]. In the current study brain gene expression levels of autopsied LOAD subjects measured from the cerebellum (n = 197) and temporal cortex (n = 202) were used. RNA extraction and gene expression measurements were previously reported [22]. Briefly, total RNA was isolated from frozen post-mortem brain tissue using the Ambion RNAqueous kit according to the manufacturer’s instructions. The quantity and quality of the RNA were evaluated using the Agilent 2100 Bioanalyzer and RNA 6000 Nano Chip. Whole Genome DASL assay (WG-DASL, Illumina, San Diego, CA) was used to measure transcript levels. This platform is designed for gene expression measurements for partially degraded RNA such as is typically isolated from frozen human brains. Details of gene expression measurements, data processing and QC were already published [22]. Briefly, 15 replicate samples measured on 5–6 different plates and on 2–3 different days were included in the study for QC and also for intra-class coefficient (ICC) [28] estimations. Raw probe level mRNA expression data were exported from GenomeStudio software (Illumina Inc.) for preprocessing with background correction, variance stabilizing transformation, quantile normalization and probe filtering using the lumi package of BioConductor [29,30]. Probes with detectable signal in >75% of the samples were used in subsequent analyses. We also annotated all of the probes by comparing their positions according to NCBI Ref Seq, Build 36.3 to those of all variants within dbSNP131 and identified the list of probes which have ≥1 variants within their sequence.

### *Genotyping*

Six *MAPT* locus haplotype tagging (ht) SNPs were selected for genotyping in the Mayo Clinic cohort (Additional file 1: Figure S1, Tables S1 and S2). SNP rs8070723 was used as a proxy for the H1/H2 haplotypes defining *del-In9*. The remaining 5 SNPs have been previously described to tag the majority of H1 sub-haplotypes [6]. Genotypes for three SNPs (rs1467967, rs242557 and rs8070723) for a subset of the samples were obtained from the Mayo Clinic LOAD GWAS (Additional file 1: Table S1). The remaining genotypes for these and all genotypes for an additional three SNPs (rs3785883, rs2471738 and rs7521) were obtained using Applied Biosystems® Taqman genotyping assays. The genotypes for these six SNPs were extracted from the ADGC GWAS data [27] using PLINK [31].

### Statistical analysis

#### MAPT single SNP association analysis with LOAD risk

All six htSNPs were tested for association with disease risk in the combined Mayo Clinic cohort, as well as individually in the JS and RS series. The same SNPs were also tested in the ADGC cohort, as well as in the ADGC + Mayo combined cohorts. All SNPs were tested for deviations from Hardy-Weinberg equilibrium (HWE) [31] in controls.

Single SNP associations with disease risk were tested assuming an additive model, using multivariable logistic regression implemented in PLINK [31] including the following covariates: Age (defined for Mayo Clinic cohort as age at diagnosis/death/last diagnosis for clinical LOAD/autopsied LOAD/controls), sex, *APOE* ϵ4 dosage and series. The analyses in the ADGC-only cohort included these covariates and also ten principal components obtained from EIGENSTRAT [32]. Mayo Clinic-only and Mayo + ADGC analyses did not include principal components, as they were not available for many of the Mayo subjects.

#### MAPT haplotype association analysis with LOAD risk

PLINK was used to estimate haplotype frequencies using the sliding window specification with a window size of six to encompass all six of the htSNPs. Haplotype associations with LOAD risk in the Mayo Clinic series were performed with both PLINK and haplo.score [33], which revealed identical results for the single haplotype analyses. According to the score statistic approach, all possible haplotypes consistent with the observed marker genotypes are obtained, maximum likelihood estimates of the haplotype frequencies, as well as the posterior probabilities of the pairs of haplotypes for each subject are computed. These posterior probabilities are then used to compute the score statistics for the association of (ambiguous) haplotypes with LOAD risk using multivariable logistic regression analysis with inclusion of the same covariates as discussed above. Only those haplotypes with frequencies >1% in the cohorts that they were tested in were included in the association analyses.

#### MAPT variant association analysis with gene expression levels

Each of the *MAPT* htSNPs and the estimated haplotypes were also tested for association with gene expression levels of *MAPT* in the TCX and CER of LOAD subjects, as measured using three probes: ILMN_1710903, ILMN_2310814 and ILMN_2298727. These LOAD subjects were also participants in our previously published eGWAS [22]. Association analysis was carried out in PLINK using linear regression approach, whereby preprocessed probe transcript levels for the three probes in each brain region (TCX and CER) were assessed as six individual quantitative phenotypes. Covariates included in the models were age at death, sex, *APOE* ϵ4 dosage, PCR plate, RNA integrity number (RIN) and adjusted RIN^2^, as described previously [22,34]. Only those haplotypes with frequencies >1% in the autopsy series that they were tested in were included in the association analyses.

## Results

### Association of MAPT single SNPs with LOAD risk

Six *MAPT* htSNPs were tested for association with LOAD risk in the Mayo Clinic and ADGC cohorts both individually and combined (Table 1). All SNPs had genotyping call rates ≥90% in the Mayo Cohort (~90-97%), ~83-100% in the ADGC cohort and ~85-100% in the combined cohort (Additional file 1: Table S2). *MAPT* rs242557 had the lowest call rate of 83% in the ADGC cohort, with all other SNPs having call rates of ≥89%. All SNPs passed the HWE cutoff of p > 1E-06 in controls, although rs242557 had HWE p < 0.05 in the Mayo Clinic, but not the ADGC controls.

**Table 1:** ***MAPT *** **single SNPs association results with LOAD risk in the Mayo, ADGC and combined Mayo + ADGC cohorts**

**SNP**	**A1**	**Mayo cohort**						**ADGC cohort**						**Mayo + ADGC**					
		**N**	**MAF_A**	**MAF_U**	**OR**	**95% CI**	**P**	**N**	**MAF_A**	**MAF_U**	**OR**	**95% CI**	**P**	**N**	**MAF_A**	**MAF_U**	**OR**	**95% CI**	**P**
rs1467967	G	**4,986**	**0.335**	**0.330**	**1.10**	**0.99 - 1.22**	**0.079**	14,365	0.329	0.327	1.05	0.99 - 1.11	0.102	**19,351**	**0.330**	**0.327**	**1.05**	**1.00 - 1.10**	**0.062**
rs242557	A	4,935	0.377	0.379	1.00	0.90 - 1.11	0.988	13,407	0.358	0.356	1.01	0.95 - 1.07	0.721	18,342	0.363	0.364	1.00	0.95 - 1.05	0.974
rs3785883	A	5,247	0.182	0.183	1.07	0.95 - 1.22	0.274	**15,187**	**0.178**	**0.171**	**1.08**	**1.01 - 1.15**	**0.032**	**20,434**	**0.179**	**0.175**	**1.06**	**1.01 - 1.13**	**0.034**
rs2471738	T	5,282	0.223	0.209	1.07	0.95 - 1.21	0.250	14,181	0.207	0.201	1.05	0.98 - 1.12	0.194	19,463	0.209	0.203	1.05	0.99 - 1.11	0.109
rs8070723	G	**5,129**	**0.209**	**0.222**	**0.81**	**0.71 - 0.91**	**7.0E-04**	**15,895**	**0.211**	**0.221**	**0.89**	**0.84 - 0.94**	**1.3E-04**	**21,024**	**0.211**	**0.221**	**0.90**	**0.85 - 0.95**	**5.3E-05**
rs7521	A	5,171	0.458	0.457	1.08	0.97 - 1.19	0.152	15,656	0.466	0.468	1.02	0.97 - 1.07	0.529	20,827	0.465	0.465	1.02	0.97 - 1.06	0.437

Results of multivariable logistic regression analyses are shown. CHR = chromosome**.** A1 = Minor Allele, N = number of subjects with genotype calls, A = Affected (LOAD subjects), U = Unaffected (Control), MAF = Minor Allele Frequency, OR = Odds Ratio, 95% CI = 95% Confidence intervals, P = p-value.

Boldface values within the tables indicate significant or suggestive associations with a p-value <0.10.

There was highly significant association of H2-tagging rs8070723-G allele with reduced risk of LOAD in the Mayo Clinic cohort (odds ratio = OR = 0.81, p = 7.0E-4) with remarkably similar OR estimates in the JS and RS series (Additional file 1: Table S3) and in the independent ADGC cohort (OR = 0.89, p = 1.3E-4) (Table 1). The association in the combined Mayo + ADGC cohort for this variant was highly significant (OR = 0.90, p = 5.3E-5) and would withstand Bonferroni correction for the six tested variants but not achieve significance at a genome-wide level.

In addition, rs3785883-A allele was nominally significant in the combined Mayo + ADGC cohort (OR = 1.06, p = 0.034) with very similar OR estimates in the Mayo Clinic (OR = 1.07, 95% confidence interval = 95%CI = 0.95-1.22) and ADGC (OR = 1.08, 95% CI = 1.01-1.15) cohorts. *MAPT* SNP rs1467967-G allele had suggestive LOAD risk association in the combined cohort (OR = 1.05, 95% CI = 1.0-1.10, p = 0.062) with risky OR estimates in both cohorts. *MAPT* SNP rs242557, previously implicated in AD [11-13] is not associated with LOAD in the combined ADGC + Mayo cohort of 18,342 subjects (p = 0.974), or the individual Mayo Clinic or ADGC cohorts.

### Association of MAPT haplotypes with LOAD risk

In the Mayo Clinic cohort of ~5,000 subjects, we identified 19 *MAPT* haplotypes with a frequency >1%. In this cohort, rs8070723-G allele tagged the H2 haplotype, present in 21.5% of the subjects, perfectly. Eighteen sub-haplotypes were identified on the H1 background. Three *MAPT* haplotypes were nominally significantly associated with LOAD risk (Table 2) and a global test for haplotypic association was also significant (p = 0.012).

**Table 2:** ***MAPT *** **haplotype association results with LOAD risk in the Mayo, ADGC and combined Mayo + ADGC cohorts**

**Haplotype**	**Alleles**	**Mayo cohort**					**ADGC cohort**					**Mayo + ADGC cohort**				
		**F_All**	**F_A**	**F_U**	**OR**	**P**	**F_All**	**F_A**	**F_U**	**OR**	**P**	**F_All**	**F_A**	**F_U**	**OR**	**P**
A (H2a)	AGGCGG	**0.215**	**0.205**	**0.221**	**0.80**	**4.1E-04**	**0.220**	**0.225**	**0.236**	**0.90**	**6.29E-04**	**0.221**	**0.228**	**0.240**	**0.90**	**1.53E-04**
B (H1b)	GGGCAA	0.173	0.180	0.169	1.15	**0.046**	0.190	0.194	0.197	1.05	0.208	**0.185**	**0.197**	**0.196**	**1.05**	**0.089**
C (H1c)	AAGTAG	0.118	0.118	0.118	0.91	0.277	0.127	0.131	0.131	1.03	0.545	0.124	0.132	0.131	1.00	0.997
D (H1d)	AAGCAA	0.076	0.071	0.078	0.99	0.905	0.075	0.074	0.079	0.93	0.195	**0.074**	**0.077**	**0.081**	**0.91**	**0.074**
E (H1e)	AGGCAA	0.074	0.075	0.073	1.12	0.308	0.077	0.082	0.081	1.00	0.961	0.077	0.083	0.081	1.02	0.618
G	GAACAA	0.017	0.014	0.018	0.91	0.692	0.012	0.013	0.013	0.84	0.256	0.014	0.014	0.015	0.85	0.176
H	AGACAA	0.044	0.038	0.048	0.91	0.506	0.042	0.045	0.041	1.08	0.309	0.042	0.045	0.045	1.03	0.641
I	GAGCAA	0.037	0.041	0.035	1.06	0.732	0.034	0.035	0.035	1.03	0.718	0.035	0.037	0.036	1.06	0.422
J	AGGCAG	0.012	0.015	0.010	1.88	**0.031**	0.010	0.011	0.010	1.20	0.292	**0.011**	**0.012**	**0.010**	**1.32**	**0.049**
L	AGACAG	0.029	0.032	0.027	1.37	**0.059**	0.032	0.034	0.033	1.06	0.483	0.031	0.034	0.032	1.10	0.187
M	GAGCAG	0.025	0.020	0.027	0.78	0.215	0.021	0.022	0.020	1.09	0.459	0.022	0.023	0.023	1.00	0.978
N	GGACAG	NA					0.011	0.011	0.011	1.12	0.527	NA				
O	AAACAA	0.016	0.016	0.017	0.90	0.696	0.018	0.020	0.018	1.18	0.192	0.018	0.020	0.019	1.09	0.457
P	GGGTAG	0.014	0.014	0.014	1.31	0.301	0.013	0.014	0.014	1.07	0.628	0.014	0.015	0.014	1.06	0.626
R	AGGTAG	0.017	0.017	0.017	1.01	0.952	0.012	0.013	0.011	1.26	0.162	0.013	0.014	0.013	1.21	0.144
U	AAGCAG	0.025	0.028	0.024	1.14	0.517	0.025	0.026	0.025	1.05	0.667	0.025	0.027	0.025	1.04	0.642
V	GGATAG	0.011	0.012	0.010	1.41	0.233	0.010	0.011	0.010	1.14	0.463	0.011	0.012	0.011	1.20	0.210
W	GGGCAG	0.012	<0.010	0.013	0.92	0.783	NA					NA				
X	GAATAG	0.016	0.018	0.014	1.59	**0.054**	0.013	0.014	0.014	1.03	0.817	0.014	0.015	0.014	1.16	0.223
Y*	AAATAG	0.015	0.017	0.014	1.64	**0.056**	0.013	0.014	0.013	1.19	0.306	0.013	0.015	0.013	1.22	0.147
Z*	GAGTAG	NA					0.011	0.011	0.010	1.10	0.590	NA				
**Global p value**		**0.0123**					**0.375**					**0.0329**				

Results of multivariable logistic regression analyses for MAPT haplotypes with frequencies >1% are shown. Haplotype nomenclature is assigned as previously reported [6,35]. Alleles for the SNPs defining the haplotypes are given in the 5’ to 3’ order as follows: rs1467967, rs242557, rs3785883, rs2471738, rs8070723, rs7521. Haplotypes not previously observed are designated by an asterisk (*). F_All = haplotype frequency in all subjects; F_A = in affected (LOAD) and F_U = unaffected (Control) subjects. OR = Odds Ratio, P = p-value.

Boldface values within the tables indicate significant or suggestive associations with a p-value <0.10.

As expected, the *MAPT* H2 haplotype was significantly associated with decreased risk for LOAD in the Mayo Clinic cohort (OR = 0.80, p = 4.1E-04). Additionally the most common sub-haplotype on the H1 background, H1b (frequency = 17.3%), was nominally significantly associated with increased risk for LOAD (OR = 1.15, p = 0.046); as was a less frequent H1 sub-haplotype J (frequency = 1.2%, OR = 1.88, p = 0.031), while three other H1 sub-haplotypes were marginally associated, also with increased LOAD risk (L, X and Y).

In the ADGC cohort, the *MAPT* H2 haplotype, was present in 22% of the subjects. On the H1 background, 19 sub-haplotypes were identified with a frequency of ≥1%. As with the Mayo Clinic cohort, H2 haplotype was significantly associated with reduced risk of LOAD in the ADGC cohort (OR = 0.90, p = 6.29E-04). None of the H1-subhaplotypes had significant association with LOAD risk in this cohort.

In the combined Mayo + ADGC cohort, there was significant global haplotypic association (p = 0.033). *MAPT* H2 haplotype had highly significant association with reduced risk of LOAD (OR = 0.90, p = 1.53E-04). *MAPT* J subhaplotype had nominally significant association with LOAD risk in the combined cohort (OR = 1.32, p = 0.049) with suggestive association observed for H1b and increased LOAD risk (OR = 1.05, p = 0.089) and for H1d and reduced LOAD risk (OR = 0.91, p = 0.074). H1c subhaplotype did not achieve significance in the Mayo Clinic, ADGC or Mayo + ADGC cohorts.

### Association of MAPT single SNPs and haplotypes with gene expression levels

In our published eGWAS [22], there were three probes on the WG-DASL platform that were used to measure *MAPT* levels: ILMN_1710903 and ILMN_2310814 that anneal to different regions of the *MAPT* 3’UTR and ILMN_2298727 that anneals to Exon 4a (Additional file 1: Figure S1). Given that the inclusion of exon 4a in tau transcripts in the central nervous system was not reported previously, we generated a quantitative PCR assay against this exon, and were able to successfully measure it in the human brain (data not shown). All three probes passed our QC threshold of detectability in >75% of subjects, with ILMN_1710903 and ILMN_2310814 detected in 100% of all AD brains tested in both the cerebellum (CER) and temporal cortex (TCX) and with ILMN_2298727 detectable in 98.0% of AD CER and 83.7% of AD TCX tissue. We previously estimated intraclass coefficients [28] for all gene expression probes, which represent the percentage of variance in expression between samples over total variance and which reflect the genetic component that contributes to variability in gene expression. We determined that both ILMN_2298727 and ILMN_1710903 had high ICC estimates of 87%, whereas ILMN_2310814 had a low ICC estimate of 18%. The variances of gene expression estimated from all subjects in our eGWAS of cerebellar tissue (n = 374) [22] revealed consistent findings for these three *MAPT* probes, with both ILMN_2298727 (0.24) and ILMN_1710903 (0.12) having variance estimates that are ~ an order of magnitude greater than that of ILMN_2310814 (0.03). We thus conclude that ILMN_2310814 is unlikely to be an informative probe.

We previously annotated all our probes for variants in their sequence [22], given the concern that such variants may result in differential binding of probes with artifactual variance in the expression levels, and therefore could result in false positive associations with genetic variants in LD with probe sequence variants [36,37]. Our annotation detected two variants within the probe sequence of ILMN_1710903 (rs67759530, rs66561280) that were also polymorphic in our autopsied AD series. ILMN_2310814 did not have any variants within its probe sequence. ILMN_2298727 annotation identified rs73314997 within its sequence, although this variant was essentially monomorphic in our eGWAS subjects [22]. Thus, of the three *MAPT* probes assessed in our gene expression analyses, ILMN_2310814 is unlikely to be informative and ILMN_1710903 may be prone to artifactual results. We therefore focused on ILMN_2298727 in our *MAPT* expression analyses (Tables 3 and 4), although we show results from all 3 *MAPT* probes for completeness.

**Table 3:** ***MAPT *** **single SNPs association results with brain *MAPT *** **gene expression levels**

**SNP**	**Brain region**	**N**	**ILMN_1710903**		**ILMN_2298727**		**ILMN_2310814**	
			**BETA**	**P**	**BETA**	**P**	**BETA**	**P**
rs1467967	CER	166	0.16	**9.3E-06**	0.11	**0.011**	0.00	0.859
	TCX	171	0.15	**2.6E-04**	0.12	**0.019**	-0.01	0.604
rs242557	CER	173	0.17	**1.2E-05**	0.08	**0.091**	0.01	0.584
	TCX	180	0.23	**3.4E-08**	0.10	**0.050**	-0.02	0.196
rs3785883	CER	175	0.14	**0.005**	0.00	0.977	-0.01	0.621
	TCX	181	0.03	0.548	-0.07	0.266	0.01	0.706
rs2471738	CER	176	0.07	0.101	0.02	0.665	0.02	0.386
	TCX	182	0.11	**0.016**	0.09	**0.089**	-0.01	0.397
rs8070723	CER	174	-0.44	**2.1E-30**	-0.16	**0.002**	0.01	0.666
	TCX	181	-0.48	**8.9E-31**	-0.20	**4.9E-04**	0.02	0.222
rs7521	CER	176	0.12	**2.8E-04**	0.08	**0.048**	0.00	0.897
	TCX	182	0.16	**1.1E-05**	0.08	**0.084**	0.00	0.976

Results of multivariable linear regression analyses are shown. Probes ILMN_1710903 and ILMN_2310814 anneal to the 3’UTR sequence and ILMN_2298727 targets exon 4a. CER = cerebellum, TCX = temporal cortex. Beta: Coefficient of association with the minor allele. P = p-value.

Boldface values within the tables indicate significant or suggestive associations with a p-value <0.10.

**Table 4:** ***MAPT *** **haplotype association results with brain *MAPT *** **gene expression levels**

**Haplotype**	**Alleles**	**Brain region**	**Freq**	**ILMN_1710903**		**ILMN_2298727**		**ILMN_2310814**	
				**BETA**	**P**	**BETA**	**P**	**BETA**	**P**
A (H2a)	AGGCGG	CER	0.212	-0.45	**8.7E-33**	-0.16	**0.003**	0.00	0.832
		TCX	0.212	-0.49	**1.1E-31**	-0.20	**0.001**	0.02	0.308
B (H1b)	GGGCAA	CER	0.180	0.15	**0.003**	0.07	0.207	-0.01	0.691
		TCX	0.180	0.13	**0.026**	0.13	**0.058**	0.01	0.630
C (H1c)	AAGTAG	CER	0.104	0.11	0.057	0.02	0.807	0.02	0.338
		TCX	0.104	0.17	**0.008**	0.05	0.536	-0.02	0.309
D (H1d)	AAGCAA	CER	0.057	0.00	0.970	0.04	0.721	0.07	0.098
		TCX	0.057	0.30	**0.007**	0.14	0.314	-0.02	0.646
E (H1e)	AGGCAA	CER	0.079	0.09	0.273	0.04	0.621	0.02	0.554
		TCX	0.079	0.15	0.079	0.13	0.212	0.00	0.964
H	AGACAA	CER	0.025	0.05	0.748	-0.13	0.478	0.04	0.538
		TCX	0.025	0.13	0.424	-0.05	0.803	-0.05	0.353
I	GAGCAA	CER	0.059	0.24	**0.010**	0.20	**0.070**	-0.04	0.322
		TCX	0.059	0.07	0.422	-0.09	0.434	-0.01	0.766
L	AGACAG	CER	0.043	0.17	0.062	0.01	0.921	-0.02	0.634
		TCX	0.043	-0.01	0.954	-0.33	**0.009**	0.01	0.841
M	GAGCAG	CER	0.028	0.26	**0.043**	0.01	0.936	0.01	0.829
		TCX	0.028	0.16	0.298	-0.23	0.204	0.00	0.927
O	AAACAA	CER	0.037	0.19	0.104	0.00	0.985	-0.04	0.435
		TCX	0.037	0.20	0.070	0.21	0.110	0.00	0.933
P	GGGTAG	CER	0.019	0.04	0.765	0.07	0.637	0.02	0.731
		TCX	0.019	0.07	0.725	0.26	0.259	-0.03	0.613
T	AGATAG	CER	0.016	0.01	0.935	-0.24	0.212	-0.07	0.285
		TCX	0.016	0.00	0.994	0.47	0.075	0.02	0.729
U	AAGCAG	CER	0.029	0.15	0.251	0.04	0.786	-0.14	**0.008**
		TCX	0.029	0.35	**0.008**	0.25	0.112	-0.01	0.836
X	GAATAG	CER	0.025	0.10	0.406	0.19	0.168	0.06	0.248
		TCX	0.025	-0.04	0.756	0.11	0.510	0.02	0.584
Y*	AAATAG	CER	0.022	-0.02	0.884	-0.13	0.458	-0.02	0.761
		TCX	0.022	-0.03	0.841	-0.03	0.882	0.00	0.933
		CER	Global p	**3.0E-42**		0.352		0.271	
		TCX		**3.2E-36**		**0.004**		0.999	

Haplotypes with frequencies > 1% are assessed with multivariable linear regression analysis. Alleles for the SNPs defining the haplotypes are given in the 5’ to 3’ order as follows: rs1467967, rs242557, rs3785883, rs2471738, rs8070723, rs7521. Haplotypes not previously observed are designated by an asterisk (*). CER = cerebellum, TCX = temporal cortex. Beta: Coefficient of association with the minor allele. P = p-value.

Boldface values within the tables indicate significant or suggestive associations with a p-value <0.10.

Evaluation of the six *MAPT* SNPs revealed significant associations between ILMN_2298727 and rs1467967, rs242557, rs8070723 and rs7521. The *MAPT* H2 haplotype tagging rs8070723 was associated with lower *MAPT* levels in both CER (β = -0.16, p = 0.002) and TCX (β = -0.20, p = 4.9E-04) of LOAD subjects (Table 3), as we previously reported in this cohort [22]. The other significant variants were associated with higher *MAPT* levels in both brain regions. Interestingly, the same variants showed associations in the same direction with the ILMN_1710903 probe, although with higher levels of significance.

Fifteen *MAPT* haplotypes with frequencies >1% were identified in the autopsied LOAD subjects with complete genotypes for the 6 variants (n = 178). There was globally significant haplotype association with the TCX gene expression levels measured with ILMN_2298727 (p = 0.004) (Table 4), that may be a reflection of the significant *MAPT* H2 association. *MAPT* H2 haplotype, as expected, was associated with lower CER (β = -0.16, p = 0.003) and TCX (β = -0.20, p = 0.001) *MAPT* levels. *MAPT* H1b was marginally associated with higher TCX levels (β = 0.13, p = 0.058), I with higher CER *MAPT* levels (β = 0.20, p = 0.07), and L with lower TCX *MAPT* levels (β = -0.33, p = 0.009). Significant associations with similar directions of effect were also observed with ILMN_1710903 and *MAPT* H2, H1b and I haplotypes.

## Discussion

In this largest to date evaluation of haplotypic variation at the *MAPT* locus in 9,814 LOAD cases and 11,550 controls, we find robust and replicable association of the *MAPT* H2 haplotype with reduced risk of LOAD or, equivalently, increased risk of LOAD with the *MAPT* H1 haplotype- in two independent cohorts from Mayo Clinic and ADGC, with similar effect size estimates. Most prior reports of haplotypic association identified LOAD risk conferred by *MAPT* H1c subhaplotype [10-12], which we were unable to replicate. One group identified an association between the *MAPT* H1 haplotype and an increased risk for amnestic mild cognitive impairment [38], which can be a prodrome to clinical AD. The only other study to evaluate *MAPT* in a large cohort (3,940 cases and 13,373 controls) also identified an association between the H2 haplotype and decreased LOAD risk [9]. In that study by Gerrish et al. [9] the H2-haplotype tagging SNP had an OR estimate of 0.89 (p = 5.20E-04), which is remarkably similar to the estimate of the H2-tagging SNP (OR = 0.90, p = 5.3E-05) and H2 haplotype (OR = 0.90, p = 1.53E-04) in our study. It should be noted that both the present study and Gerrish et al. included samples from the ADNI and TGen series. We confirmed that the *MAPT* H2 association retains its significance in the ADGC cohort even after removal of these two datasets (OR = 0.87, p = 6.1E-04). Thus, there is evidence of *MAPT* H2 association with reduced risk of LOAD in two large and independent studies. Though robust, this LOAD risk association does not achieve genome wide significance in either study or a p value < 1.0E-7 in the recent meta-analysis of 74,046 individuals by the IGAP consortium [39]. It will be important to evaluate the IGAP dataset for availability of *MAPT* haplotype tagging variants and to pursue an in-depth analysis of haplotypic association at this locus.

Although the *MAPT* H2 haplotypic association with LOAD was clearly the strongest of the *MAPT* haplotypes and one that we previously reported [22], we identified additional SNPs and haplotypes with nominal significance in our study. These weaker associations would not withstand multiple testing and could represent false positives and require replication in additional series. It should be noted that some of these variants, such as rs3785883, H1b, H1d and J showed consistent direction of effect in the Mayo Clinic and ADGC cohorts. *MAPT* rs3785883 minor allele was previously shown to associate with higher levels of CSF tau, phospho-tau and earlier age at onset [16]. Although this prior smaller study did not identify association with LOAD risk, the biological effect of this variant which associates with increased LOAD risk in our study appears to be consistent between these two studies.

We and others previously reported association between *MAPT* haplotypes and brain *MAPT* levels [11,19-22]. In this study, we evaluated *MAPT* subhaplotypes for association with brain *MAPT* levels in two brain regions from LOAD subjects. The most robust gene expression association occurs with the H2 haplotype, as we had reported [22] (β_CER_ = -0.16, p_CER_ = 0.003; β_TCX_ = -0.20, p_TCX_ = 0.001), that also has the strongest association with LOAD risk in our study. We find that this haplotype with a protective effect on LOAD associates with lower brain *MAPT* levels. Given multiple *MAPT* alternatively spliced exons leading to multiple transcripts, each with potentially different effects on function [1,20], uncovering the precise regulatory change associated with genotypic variation in this region is critical. In our study, we mainly focus on results from one probe, ILMN_2298727, that is both informative and does not have a variant in its sequence based on annotation and genotyping. This probe is expected to anneal to exon 4a, however the expression levels obtained from it can be a surrogate for total *MAPT* levels or levels of any of the alternatively spliced exon-containing transcripts that reside with exon 4a. Indeed, gene expression associations with this probe are consistent with those from ILMN_1710903, which should recognize all transcripts, although ILMN_1710903 is confounded by a confirmed variant within its sequence. Our findings are also congruous with prior reports of associations of H1 haplotype or H1c sub-haplotype with higher 4R [19] and or total *MAPT*[11] levels, as measured by alternative gene expression measurement methods.

We did not identify significant associations between the H1c subhaplotype and brain *MAPT* levels, though we did observe suggestive associations between both CER and TCX *MAPT* levels and rs242557, a variant that partially tags H1c. *MAPT* rs1467967 associated with significant *MAPT* elevations in both brain regions and a suggestive association with LOAD risk, which is biologically consistent. These and additional weaker gene expression associations with variants such as H1b, I, L and rs7521 requires further replications.

In summary, our study provides evidence of robust LOAD risk and brain *MAPT* level associations with *MAPT* H2 haplotype and nominates additional variants and subhaplotypes for further investigations in LOAD. The overall genetic contribution of *MAPT* variants to LOAD risk appears to be modest, in contrast to primary tauopathies, where the H1 haplotype, for example, has an estimated OR of 5.5 from the PSP GWAS [8]. This may be due to different sets of functional variants residing in the same haplotypic backbone and leading to different biological outcomes resulting either in a primary tauopathy vs. tau pathology in LOAD; a more complex genetic architecture in LOAD with contribution from multiple functional variants in different pathways; or a combination of both. Discovering the precise sets of *MAPT* functional variants; and assessing their biologic consequence, especially on transcriptional regulation, may be critical to deciphering the commonalities and distinctions in the etiology of LOAD vs. primary tauopathies. Our study highlights the importance of in-depth association of *MAPT* haplotypic variation in well-powered cohorts and nominates H2 and additional variants as LOAD risk factors with effects on gene expression. Larger scale *MAPT* haplotype LOAD risk association studies, variant discovery efforts targeting specific haplotypes and transcriptional studies that jointly evaluate haplotypes and specific transcripts are warranted.

## Conclusions

In summary, these findings confirm associations between *MAPT* H2 haplotype and both reduced risk of LOAD and lower *MAPT* transcript brain levels. In addition, we describe additional *MAPT* variants and subhaplotypes that associate with LOAD risk and/or brain *MAPT* levels, which require confirmation in additional series. These results highlight the importance of joint utilization of gene expression and disease risk phenotypes. Additionally, these biologically consistent findings should encourage screening efforts in the *MAPT* region for discovery of regulatory variants that confer LOAD risk via influencing brain levels of *MAPT* transcripts.

## Abbreviations

Aβ: Amyloid-beta; AD: Alzheimer’s disease; ADGC: Alzheimer’s disease genetics consortium; CBD: Corticobasal degeneration; CSF: Cerebrospinal fluid; CER: Cerebellum; eGWAS: Gene expression genome-wide association study; GWAS: Genome-wide association study; HWE: Hardy-Weinberg equilibrium; ICC: Intraclass coefficient; JS: Mayo Clinic Jacksonville, FL series; LD: Linkage disequilibrium; LOAD: Late-onset Alzheimer’s disease; MAPT: Microtubule-associated protein tau; NFT: Neurofibrillary tangles; OR: Odds ratio; PSP: Progressive supranuclear palsy; QC: Quality control; RS: Mayo Clinic Rochester, MN series; SNP: Single nucleotide polymorphism; TCX: Temporal cortex.

## Competing interests

R.C. Petersen, M.D., Ph.D. has been a consultant to GE Healthcare and Elan Pharmaceuticals, has served on a data safety monitoring board in clinical trials sponsored by Pfizer Incorporated and Janssen Alzheimer Immunotherapy and gave a CME lecture at Novartis Incorporated. N. Graff-Radford, M.D. has served as a consultant to Codman and received grant support from Elan Pharmaceutical Research, Pfizer Pharmaceuticals, Medivation, and Forrest.

## Authors’ contributions

MA conceived and designed the experiments, performed the experiments, analyzed the data and wrote the paper, MK performed the experiments, ZQ performed the experiments, FZ performed the experiments, HSC analyzed the data, CSY analyzed the data, JC analyzed the data, VSP analyzed the data, MMC performed the experiments, MMC performed the experiments, SK performed the experiments, TN performed the experiments, LM performed the experiments, KGM performed the experiments, SL performed the experiments, GB performed the experiments, CPK performed the experiments, JJ performed the experiments, SM contributed reagents/materials/analysis tools, JKK contributed reagents/materials/analysis tools, PKC contributed reagents/materials/analysis tools, JLH contributed reagents/materials/analysis tools, RM contributed reagents/materials/analysis tools, MAPV contributed reagents/materials/analysis tools, LAF contributed reagents/materials/analysis tools, GDS contributed reagents/materials/analysis tools, ADGC contributed reagents/materials/analysis tools, JEP contributed reagents/materials/analysis tools, RCP contributed reagents/materials/analysis tools, NRGR contributed reagents/materials/analysis tools, DWD contributed reagents/materials/analysis tools, SGY contributed reagents/materials/analysis tools, NET conceived and designed the experiments, analyzed the data and wrote the paper. All authors read and approved the final manuscript.

## ADGC acknowledgements

The National Institutes of Health, National Institute on Aging (NIH-NIA) supported this work through the following grants: ADGC, U01 AG032984, RC2 AG036528; NACC, U01 AG016976; NCRAD, U24 AG021886; NIA LOAD, U24 AG026395, U24 AG026390; Banner Sun Health Research Institute P30 AG019610; Boston University, P30 AG013846, U01 AG10483, R01 CA129769, R01 MH080295, R01 AG017173, R01 AG025259, R01AG33193; Columbia University, P50 AG008702, R37 AG015473; Duke University, P30 AG028377, AG05128; Emory University, AG025688; Group Health Research Institute, UO1 AG06781, UO1 HG004610; Indiana University, P30 AG10133; Johns Hopkins University, P50 AG005146, R01 AG020688; Massachusetts General Hospital, P50 AG005134; Mayo Clinic, P50 AG0016574, U01 AG006786; Mount Sinai School of Medicine, P50 AG005138, P01 AG002219; New York University, P30 AG08051, MO1RR00096, UL1 RR029893, 5R01AG012101, 5R01AG022374, 5R01AG013616, 1RC2AG036502, 1R01AG035137; Northwestern University, P30 AG013854; Oregon Health & Science University, P30 AG008017, R01 AG026916; Rush University, P30 AG010161, R01 AG019085, R01 AG15819, R01 AG17917, R01 AG30146; TGen, R01 NS059873; University of Alabama at Birmingham, P50 AG016582, UL1RR02777; University of Arizona, R01 AG031581; University of California, Davis, P30 AG010129; University of California, Irvine, P50 AG016573, P50, P50 AG016575, P50 AG016576, P50 AG016577; University of California, Los Angeles, P50 AG016570; University of California, San Diego, P50 AG005131; University of California, San Francisco, P50 AG023501, P01 AG019724; University of Kentucky, P30 AG028383, AG05144; University of Michigan, P50 AG008671; University of Pennsylvania, P30 AG010124; University of Pittsburgh, P50 AG005133, AG030653; University of Southern California, P50 AG005142; University of Texas Southwestern, P30 AG012300; University of Miami, R01 AG027944, AG010491, AG027944, AG021547, AG019757; University of Washington, P50 AG005136; Vanderbilt University, R01 AG019085; and Washington University, P50 AG005681, P01 AG03991. The Kathleen Price Bryan Brain Bank at Duke University Medical Center is funded by NINDS grant # NS39764, NIMH MH60451 and by Glaxo Smith Kline. Genotyping of the TGEN2 cohort was supported by Kronos Science. The TGen series was also funded by NIA grant AG034504 to AJM, The Banner Alzheimer’s Foundation, The Johnnie B. Byrd Sr. Alzheimer’s Institute, the Medical Research Council, and the state of Arizona and also includes samples from the following sites: Newcastle Brain Tissue Resource (funding via the Medical Research Council, local NHS trusts and Newcastle University), MRC London Brain Bank for Neurodegenerative Diseases (funding via the Medical Research Council),South West Dementia Brain Bank (funding via numerous sources including the Higher Education Funding Council for England (HEFCE), Alzheimer’s Research Trust (ART), BRACE as well as North Bristol NHS Trust Research and Innovation Department and DeNDRoN), The Netherlands Brain Bank (funding via numerous sources including Stichting MS Research, Brain Net Europe, Hersenstichting Nederland Breinbrekend Werk, International Parkinson Fonds, Internationale Stiching Alzheimer Onderzoek), Institut de Neuropatologia, Servei Anatomia Patologica, Universitat de Barcelona. ADNI Funding for ADNI is through the Northern California Institute for Research and Education by grants from Abbott, AstraZeneca AB, Bayer Schering Pharma AG, Bristol-Myers Squibb, Eisai Global Clinical Development, Elan Corporation, Genentech, GE Healthcare, GlaxoSmithKline, Innogenetics, Johnson and Johnson, Eli Lilly and Co., Medpace, Inc., Merck and Co., Inc., Novartis AG, Pfizer Inc, F. Hoffman-La Roche, Schering-Plough, Synarc, Inc., Alzheimer's Association, Alzheimer's Drug Discovery Foundation, the Dana Foundation, and by the National Institute of Biomedical Imaging and Bioengineering and NIA grants U01 AG024904, RC2 AG036535, K01 AG030514. We thank Drs. D. Stephen Snyder and Marilyn Miller from NIA who are *ex-officio* ADGC members. Support was also from the Alzheimer’s Association (LAF, IIRG-08-89720; MP-V, IIRG-05-14147) and the US Department of Veterans Affairs Administration, Office of Research and Development, Biomedical Laboratory Research Program. P.S.G.-H. is supported by Wellcome Trust, Howard Hughes Medical Institute, and the Canadian Institute of Health Research.

## ADGC affiliations

^1^Department of Neurology, Johns Hopkins University, Baltimore, Maryland, ^2^Department of Neurology, University of Michigan, Ann Arbor, Michigan, ^3^Geriatric Research, Education and Clinical Center (GRECC), VA Ann Arbor Healthcare System (VAAAHS), Ann Arbor, Michigan, ^4^Michigan Alzheimer Disease Center, Ann Arbor, Michigan, ^5^Department of Neurology, University of California Los Angeles, Los Angeles, California, ^6^Department of Psychiatry, University of Pennsylvania Perelman School of Medicine, Philadelphia, Pennsylvania, ^7^Geriatric Research, Education and Clinical Center (GRECC), University of Wisconsin, Madison, Wisconsin, ^8^Department of Medicine, University of Wisconsin, Madison, Wisconsin, ^9^Wisconsin Alzheimer's Institute, Madison, Wisconsin, ^10^Department of Medicine (Genetics Program), Boston University, Boston, Massachusetts, ^11^Department of Pharmacology and Neuroscience, University of North Texas Health Science Center, Fort Worth, Texas, ^12^Department of Human Genetics, University of Pittsburgh, Pittsburgh, Pennsylvania, ^13^Department of Neurological Sciences, Rush University Medical Center, Chicago, Illinois, ^14^Department of Behavioral Sciences, Rush University Medical Center, Chicago, Illinois, ^15^Civin Laboratory for Neuropathology, Banner Sun Health Research Institute, Phoenix, Arizona, ^16^Departments of Psychiatry, Neurology, and Psychology, University of Pittsburgh School of Medicine, Pittsburgh, Pennsylvania, ^17^The John P. Hussman Institute for Human Genomics, University of Miami, Miami, Florida, ^18^Dr. John T. Macdonald Foundation Department of Human Genetics, University of Miami, Miami, Florida, ^19^National Alzheimer's Coordinating Center, University of Washington, Seattle, Washington, ^20^Rush Alzheimer's Disease Center, Rush University Medical Center, Chicago, Illinois, ^21^Department of Pathology, Northwestern University Feinberg School of Medicine, Chicago, Illinois, ^22^Cognitive Neurology and Alzheimer's Disease Center, Northwestern University Feinberg School of Medicine, Chicago, Illinois, ^23^Department of Neurology, University of Washington, Seattle, Washington, ^24^VA Puget Sound Health Care System/GRECC, Seattle, Washington, ^25^Department of Epidemiology, Harvard School of Public Health, Boston, Massachusetts, ^26^Department of Psychiatry, Massachusetts General Hospital/Harvard Medical School, Boston, Massachusetts, ^27^Department of Neurology, Mayo Clinic, Rochester, Minnesota, ^28^Swedish Medical Center, Seattle, Washington, ^29^Department of Neurology, University of California San Francisco, San Francisco, California, ^30^Department of Medicine, Duke University, Durham, North Carolina, ^31^Department of Neuroscience, Mount Sinai School of Medicine, New York, New York, ^32^Department of Psychiatry, Mount Sinai School of Medicine, New York, New York, ^33^Departments of Genetics and Genomic Sciences, Mount Sinai School of Medicine, New York, New York, ^34^Department of Pathology and Immunology, Washington University, St. Louis, Missouri, ^35^Department of Pathology and Laboratory Medicine, University of Pennsylvania Perelman School of Medicine, Philadelphia, Pennsylvania, ^36^USF Health Byrd Alzheimer's Institute, University of South Florida, Tampa, Florida, ^37^Fred Hutchinson Cancer Research Center, Seattle, Washington, ^38^Department of Psychiatry and Behavioral Sciences, Miller School of Medicine, University of Miami, Miami, Florida, ^39^Department of Pathology, University of Alabama at Birmingham, Birmingham, Alabama, ^40^Department of Neurology, University of Southern California, Los Angeles, California, ^41^Department of Neurology, University of Alabama at Birmingham, Birmingham, Alabama, ^42^Neurogenomics Division, Translational Genomics Research Institute, Phoenix, Arizona, ^43^Department of Medicine, University of Washington, Seattle, Washington, ^44^Department of Neurology, University of California Irvine, Irvine, California, ^45^Department of Psychiatry and Hope Center Program on Protein Aggregation and Neurodegeneration, Washington University School of Medicine, St. Louis, Missouri, ^46^Program in Translational NeuroPsychiatric Genomics, Institute for the Neurosciences, Department of Neurology & Psychiatry, Brigham and Women's Hospital and Harvard Medical School, Boston, Massachusetts, ^47^Program in Medical and Population Genetics, Broad Institute, Cambridge, Massachusetts, ^48^Department of Neurology, University of California Davis, Sacramento, California, ^49^University of Virginia School of Medicine, Charlottesville, Virginia, ^50^Institute for Memory Impairments and Neurological Disorders, University of California Irvine, Irvine, California, ^51^Wien Center for Alzheimer's Disease and Memory Disorders, Mount Sinai Medical Center, Miami Beach, Florida, ^52^Rush Institute for Healthy Aging, Department of Internal Medicine, Rush University Medical Center, Chicago, Illinois, ^53^Department of Medical and Molecular Genetics, Indiana University, Indianapolis, Indiana, ^54^Department of Neurology, Indiana University, Indianapolis, Indiana, ^55^Department of Psychiatry, New York University, New York, New York, ^56^C.S. Kubik Laboratory for Neuropathology, Massachusetts General Hospital, Charlestown, Massachusetts, ^57^Department of Neurosciences, University of California San Diego, La Jolla, California, ^58^Department of Psychiatry, University of Pittsburgh, Pittsburgh, Pennsylvania, ^59^Department of Pathology and Laboratory Medicine, Emory University, Atlanta, Georgia, ^60^Emory Alzheimer's Disease Center, Emory University, Atlanta, Georgia, ^61^Neurogenetics Program, University of California Los Angeles, Los Angeles, California, ^62^Department of Pathology and Laboratory Medicine, Indiana University, Indianapolis, Indiana, ^63^Department of Neurology, Emory University, Atlanta, Georgia, ^64^Division of Genetics, Department of Medicine and Partners Center for Personalized Genetic Medicine, Brigham and Women's Hospital and Harvard Medical School, Boston, Massachusetts, ^65^Department of Neurology, Massachusetts General Hospital/Harvard Medical School, Boston, Massachusetts, ^66^Center for Applied Genomics, Children's Hospital of Philadelphia, Philadelphia, Pennsylvania, ^67^Department of Pathology (Neuropathology), University of Pittsburgh, Pittsburgh, Pennsylvania, ^68^Institute of Neurology, University College London, Queen Square, London, ^69^Sanders-Brown Center on Aging, Department of Molecular and Biomedical Pharmacology, University of Kentucky, Lexington, Kentucky, ^70^Taub Institute on Alzheimer's Disease and the Aging Brain, Department of Neurology, Columbia University, New York, New York, ^71^Department of Pathology, Duke University, Durham, North Carolina, ^72^Department of Genome Sciences, University of Washington, Seattle, Washington, ^73^Department of Medicine (Medical Genetics), University of Washington, Seattle, Washington, ^74^Sanders-Brown Center on Aging, Department Neurology, University of Kentucky, Lexington, Kentucky, ^75^Department of Pathology and Laboratory Medicine, University of California Davis, Sacramento, California, ^76^Department of Biostatistics, Boston University, Boston, Massachusetts, ^77^Department of Ophthalmology, Boston University, Boston, Massachusetts, ^78^University of Pittsburgh Alheimer's Disease Research Center, Pittsburgh, Pennsylvania, ^79^Department of Neurology, Oregon Health & Science University, Portland, Oregon, ^80^Department of Neurology, Portland Veterans Affairs Medical Center, Portland, Oregon, ^81^Department of Pathology and Laboratory Medicine, University of California Irvine, Irvine, California, ^82^Department of Neurology, Boston University, Boston, Massachusetts, ^83^Department of Pathology, Boston University, Boston, Massachusetts, ^84^Department of Neuropsychology, University of California San Francisco, San Francisco, California, ^85^Department of Molecular & Medical Genetics, Oregon Health & Science University, Portland, Oregon, ^86^Department of Epidemiology, University of Washington, Seattle, Washington, ^87^Department of Neurobiology and Behavior, University of California Irvine, Irvine, California, ^88^Group Health Research Institute, Group Health, Seattle, Washington, ^89^Department of Pathology, University of Washington, Seattle, Washington, ^90^Department of Psychiatry and Behavioral Sciences, University of Washington School of Medicine, Seattle, Washington, ^91^Department of Pathology, University of Michigan, Ann Arbor, Michigan, ^92^Department of Psychiatry, Johns Hopkins University, Baltimore, Maryland, ^93^Department of Preventive Medicine, University of Southern California, Los Angeles, California, ^94^Department of Medicine - Pulmonary, New York University, New York, New York, ^95^Department of Neurology, University of Miami, Miami, Florida, ^96^Department of Pathology, University of California San Diego, La Jolla, California, ^97^School of Nursing Northwest Research Group on Aging, University of Washington, Seattle, Washington, ^98^Department of Neurology, Northwestern University Feinberg School of Medicine, Chicago, Illinois, ^99^Department of Pathology, University of Southern California, Los Angeles, California, ^100^Department of Neurology, Washington University, St. Louis, Missouri, ^101^Arizona Alzheimer’s Consortium, Phoenix, Arizona, ^102^Department of Psychiatry, University of Arizona, Phoenix, Arizona, ^103^Banner Alzheimer's Institute, Phoenix, Arizona, ^104^Alzheimer's Disease Center, New York University, New York, New York, ^105^Gertrude H. Sergievsky Center, Columbia University, New York, New York, ^106^Department of Neurology, Columbia University, New York, New York, ^107^Tanz Centre for Research in Neurodegenerative Disease, University of Toronto, Toronto, Ontario, ^108^Department of Neurology, University of Texas Southwestern, Dallas, Texas, ^109^Department of Radiology and Imaging Sciences, Indiana University, Indianapolis, Indiana, ^110^Department of Pathology (Neuropathology), Rush University Medical Center, Chicago, Illinois, ^111^Department of Psychiatry, University of Southern California, Los Angeles, California, ^112^Cambridge Institute for Medical Research and Department of Clinical Neurosciences, University of Cambridge, Cambridge, ^113^Center for Human Genetics and Research, Department of Molecular Physiology and Biophysics, Vanderbilt University, Nashville, Tennessee, ^114^Department of Pathology, Johns Hopkins University, Baltimore, Maryland, ^115^Sanders-Brown Center on Aging, Department of Anatomy and Neurobiology, University of Kentucky, Lexington, Kentucky, ^116^Department of Pathology & Laboratory Medicine, University of California Los Angeles, Los Angeles, California, ^117^Taub Institute on Alzheimer's Disease and the Aging Brain, Department of Pathology, Columbia University, New York, New York, ^118^Department of Psychiatry, Northwestern University Feinberg School of Medicine, Chicago, Illinois, ^119^Department of Psychiatry & Behavioral Sciences, Duke University, Durham, North Carolina, ^120^Department of Pathology, Oregon Health & Science University, Portland, Oregon, ^121^Evelyn F. McKnight Brain Institute, Department of Neurology, Miller School of Medicine, University of Miami, Miami, Florida.

## ADGC co-authors

Marilyn S. Albert^1^, Roger L. Albin^2–4^, Liana G. Apostolova^5^, Steven E. Arnold^6^, Sanjay Asthana^7–9^, Craig S. Atwood^7,9^, Clinton T. Baldwin^10^, Robert Barber^11^, Michael M. Barmada^12^, Lisa L. Barnes^13,14^, Thomas G. Beach^15^, James T. Becker^16^, Gary W. Beecham^17,18^, Duane Beekly^19^, David A. Bennett^13,20^, Eileen H. Bigio^21,22^, Thomas D. Bird^23,24^, Deborah Blacker^25,26^, Bradley F. Boeve^27^, James D. Bowen^28^, Adam Boxer^29^, James R. Burke^30^, Joseph D. Buxbaum^31–33^, Nigel J. Cairns^34^, Laura B. Cantwell^35^, Chuanhai Cao^36^, Chris S. Carlson^37^, Cynthia M. Carlsson^8^, Regina M. Carney^38^, Steven L. Carroll^39^, Helena C. Chui^40^, David G. Clark^41^, Jason Corneveaux^42^, David H. Cribbs^44^, Elizabeth A. Crocco^38^, Carlos Cruchaga^45^, Philip L. De Jager^46,47^, Charles DeCarli^48^, Steven T. DeKosky^49^, F. Yesim Demirci^12^, Malcolm Dick^50^, Ranjan Duara^51^, Denis Evans^52^, Kelley M. Faber^53^, Kenneth B. Fallon^39^, Martin R. Farlow^54^, Steven Ferris^55^, Tatiana M. Foroud^53^, Matthew P. Frosch^56^, Douglas R. Galasko^57^, Mary Ganguli^58^, Marla Gearing^59,60^, Daniel H. Geschwind^61^, Bernardino Ghetti^62^, John R. Gilbert^17,18^, Jonathan D. Glass^63^, Alison M. Goate^45^, Robert C. Green^64^, John H. Growdon^65^, Hakon Hakonarson^66^, Ronald L. Hamilton^67^, Kara L. Hamilton-Nelson^17^, John Hardy^68^, Lindy E. Harrell^41^, Elizabeth Head^69^, Lawrence S. Honig^70^, Matthew J. Huentelman^42^, Christine M. Hulette^71^, Bradley T. Hyman^65^, Gail P. Jarvik^72,73^, Gregory A. Jicha^74^, Lee-Way Jin^75^, Gyungah Jun^10,76,77^, M. Ilyas Kamboh^12,78^, Anna Karydas^29^, Jeffrey A. Kaye^79,80^, Ronald Kim^81^, Edward H. Koo^57^, Neil W. Kowall^82,83^, Joel H. Kramer^84^, Patricia Kramer^79,85^, Walter A. Kukull^86^, Brian W. Kunkle^17^, Frank M. LaFerla^87^, James J. Lah^63^, Eric B. Larson^43,88^, James B. Leverenz^89^, Allan I. Levey^63^, Ge Li^90^, Andrew P. Lieberman^91^, Chiao-Feng Lin^35^, Oscar L. Lopez^78^, Kathryn L. Lunetta^76^, Constantine G. Lyketsos^92^, Wendy J. Mack^93^, Daniel C. Marson^41^, Eden R. Martin^17,18^, Frank Martiniuk^94^, Deborah C. Mash^95^, Eliezer Masliah^57,96^, Wayne C. McCormick^43^, Susan M. McCurry^97^, Andrew N. McDavid^37^, Ann C. McKee^82,83^, Marsel Mesulam^22,98^, Bruce L. Miller^29^, Carol A. Miller^99^, Joshua W. Miller^75^, Thomas J. Montine^89^, John C. Morris^34,100^, Jill R. Murrell^53,62^, Amanda J. Myers^38^, Adam C. Naj^35^, John M. Olichney^48^, Amanda Partch^35^, Henry L. Paulson^2^, William Perry^17^, Elaine Peskind^90^, Aimee Pierce^44^, Wayne W. Poon^50^, Huntington Potter^36^, Joseph F. Quinn^79^, Ashok Raj^36^, Murray Raskind^90^, Eric M. Reiman^42,101–103^, Barry Reisberg^55,104^, Christiane Reitz^70,105,106^, John M. Ringman^5^, Erik D. Roberson^41^, Ekaterina Rogaeva^107^, Howard J. Rosen^29^, Roger N. Rosenberg^108^, Mark A. Sager^8^, Mary Sano^32^, Andrew J. Saykin^53,109^, Julie A. Schneider^13,110^, Lon S. Schneider^40,111^, William W. Seeley^29^, Amanda G. Smith^36^, Joshua A. Sonnen^89^, Salvatore Spina^62^, Peter St George-Hyslop^107,112^, Robert A. Stern^82^, Rudolph E. Tanzi^65^, Tricia A. Thornton-Wells^113^, John Q. Trojanowski^35^, Juan C. Troncoso^114^, Debby W. Tsuang^24,90^, Otto Valladares^35^, Vivianna M. Van Deerlin^35^, Linda J. Van Eldik^115^, Badri N. Vardarajan^70,105,106^, Harry V. Vinters^5,116^, Jean Paul Vonsattel^117^, Li-San Wang^35^, Sandra Weintraub^22,118^, Kathleen A. Welsh-Bohmer^30,119^, Jennifer Williamson^70^, Sarah Wishnek^17^, Randall L. Woltjer^120^, Clinton B. Wright^121^, Chang-En Yu^43^, Lei Yu^13^.

## ADGC disclosure statement

T.D.B. received licensing fees from and is on the speaker's bureau of Athena Diagnostics, Inc. M.R.F. receives research funding from BristolMyersSquibb Company, Danone Research, Elan Pharmaceuticals, Inc., Eli Lilly and Company, Novartis Pharmaceuticals Corporation, OctaPharma AG, Pfizer Inc., and Sonexa Therapeutics, Inc; Receives honoraria as scientific consultant from Accera, Inc., Astellas Pharma US Inc., Baxter, Bayer Pharmaceuticals Corporation, BristolMyersSquibb, Eisai Medical Research, Inc., GE Healthcare, Medavante, Medivation, Inc., Merck & Co., Inc., Novartis Pharmaceuticals Corp., Pfizer, Inc., Prana Biotechnology Ltd., QR Pharma., Inc., The sanofi-aventis Group, and Toyama Chemical Co., Ltd.; and is speaker for Eisai Medical Research, Inc., Forest Laboratories, Pfizer Inc. and Novartis Pharmaceuticals Corporation. A.M.G. has research funding from AstraZeneca, Pfizer and Genentech, and has received remuneration for giving talks at Pfizer and Genentech. R.C.P. is on the Safety Monitory Committee of Pfizer, Inc. (Wyeth) and a consultant to the Safety Monitoring Committee at Janssen Alzheimer's Immunotherapy Program (Elan), to Elan Pharmaceuticals, and to GE Healthcare. R.E.T. is a consultant to Eisai, Japan in the area of Alzheimer's genetics and a shareholder in, and consultant to Pathway Genomics, Inc, San Diego, CA.

## Supplementary Material

Additional file 1 **This file includes Table S1.** (Demographic information of the cohorts); **Table S2.** (Genotype counts, call rates and Hardy Weinberg results); **Table S3.** (*MAPT* single SNP association results with LOAD risk in the individual Mayo Clinic series. Results of multivariable logistic regression analysis); **Figure S1.** (*MAPT* Refseq mRNA isoforms and SNP annotation). Click here for file
