# Supplementary material for: Association of MAPT haplotypes with Alzheimer’s disease risk and MAPT brain gene expression levels
Source: Alzheimers Res Ther. 2014 Jul 1;6(4):39. doi: 10.1186/alzrt268 (PMC4198935; doi:10.1186/alzrt268)
Supplement: Additional file 1 — This file includes Table S1. (Demographic information of the cohorts); Table S2. (Genotype counts, call rates and Hardy Weinberg results); Table S3. (MAPT single SNP association results with LOAD risk in the individual Mayo Clinic series. Results of multivariable logistic regression analysis); Figure S1. (MAPT Refseq mRNA isoforms and SNP annotation). [file alzrt268-S1.docx]

**Supplementary File**

**Association of *MAPT* haplotypes with Alzheimer’s disease risk and *MAPT* brain gene expression levels.**

Mariet Allen, Ph.D.^1^, Michaela Kachadoorian^1^, Zachary Quicksall^1^, Fanggeng Zou, Ph.D.^1^, High Seng Chai, Ph.D.^2^, Curtis Younkin^1^, Julia E. Crook, Ph.D.^3^, V. Shane Pankratz, Ph.D.^2^, Minerva M. Carrasquillo, Ph.D.^1^, Siddharth Krishnan^1^, Thuy Nguyen^1^, Li Ma^1^, Kimberly Malphrus^1^, Sarah Lincoln^1^, Gina Bisceglio^1^, Christopher P. Kolbert^4^, Jin Jen, Ph.D.^4^, Shubhabrata Mukherjee, Ph.D.^5^, John K. Kauwe, Ph.D.^6^, Paul K. Crane, M.D., M.P.H.^5^, Jonathan L. Haines, Ph.D.^7,8^, Richard Mayeux, M.D.^9^, Margaret A. Pericak-Vance, Ph.D.^10^, Lindsay A. Farrer, Ph.D.^11^, Gerard D. Schellenberg, Ph.D.^12^, Alzheimer’s Disease Genetics Consortium (ADGC)#, Joseph E. Parisi, M.D.^13^, Ronald C. Petersen, M.D., Ph.D.^14^, Neill R. Graff-Radford, M.D.^15^, Dennis W. Dickson, M.D.^1^, Steven G. Younkin, M.D., Ph.D.^1^, Nilüfer Ertekin-Taner, M.D., Ph.D.^1,15*^

**Supplementary Table 1.**

| **Series** | **LOAD** | | | | **Control** | | | |
| --- | --- | --- | --- | --- | --- | --- | --- | --- |
|  | **N** | **% Female** | **Mean Age (SD)** | **N GWAS** | **N** | **% Female** | **Mean Age (SD)** | **N GWAS** |
| JS | 886 | 62% | 77.7 (6.5) | 340 | 981 | 58% | 76.5 (7.4) | 328 |
| RS | 615 | 61% | 80.0 (7.8) | 240 | 2425 | 54% | 78.3 (5.6) | 678 |
| AUT | 551 | 59% | 81.2 (8.6) | 224 | NA | | | |
| Mayo Cohort | 2052 | 61% | 79.3 (7.6) | 804 | 3406 | 55% | 77.8 (6.2) | 1,006 |
| ADGC Cohort | 7,762 | 59% | 75.7 (8.2) | NA | 8,144 | 60% | 77.7 (8.2) | NA |

**Supplementary Table 1.** **Demographic information of the cohorts**. JS = Jacksonville Series, RS = Rochester Series, AUT = Autopsied LOAD subjects, Mayo Cohort = JS+RS+AUT series combined, ADGC = Alzheimer’s Disease Genetics Consortium. N=Number of Subjects, SD-Standard Deviation, N GWAS = Number of subjects included in the Mayo GWAS published study[^1^](#_ENREF_1). NA= not available. Mean age of diagnosis for clinical LOAD, death for autopsied LOAD and last evaluation for control subjects.

**Supplementary Table 2.**

| **SNP** | **A1** | **Mayo Cohort** | | | | **ADGC Cohort** | | | | **JS** | | | **RS** | | | **AUT** |
| --- | --- | --- | --- | --- | --- | --- | --- | --- | --- | --- | --- | --- | --- | --- | --- | --- |
|  |  | **Geno-A** | **Geno-U** | **HWE-U** | **Call Rates** | **Geno-A** | **Geno-U** | **HWE-U** | **Call Rates** | **Geno-A** | **Geno-U** | **HWE-U** | **Geno-A** | **Geno-U** | **HWE-U** | **Geno-A** |
| rs1467967 | G | 220/812/836 | 340/1376/1402 | 0.935 | 0.91 | 765/3151/3194 | 752/3232/3271 | 0.274 | 0.89 | 91/372/368 | 85/408/412 | 0.285 | 70/241/225 | 255/968/990 | 0.445 | 59/199/243 |
| rs242557 | A | 260/838/704 | 500/1373/1260 | 1.25E-04 | 0.90 | 865/3082/2758 | 849/3081/2772 | 0.894 | 0.83 | 124/386/318 | 141/404/387 | 0.041 | 60/215/185 | 359/969/873 | 0.001 | 76/237/201 |
| rs3785883 | A | 66/581/1307 | 110/982/2201 | 0.954 | 0.96 | 254/2135/5008 | 235/2203/5352 | 0.661 | 0.96 | 30/238/573 | 26/267/650 | 0.908 | 23/176/379 | 84/715/1551 | 0.892 | 13/167/355 |
| rs2471738 | T | 106/671/1203 | 135/1112/2055 | 0.344 | 0.97 | 292/2265/4385 | 287/2315/4637 | 0.971 | 0.89 | 39/297/515 | 28/334/585 | 0.017 | 33/194/358 | 107/778/1470 | 0.757 | 34/180/330 |
| rs8070723 | G | 82/630/1191 | 151/1127/1948 | 0.475 | 0.94 | 373/2533/4851 | 394/2814/4930 | 0.797 | 1.00 | 35/287/516 | 49/354/536 | 0.372 | 17/172/363 | 102/773/1412 | 0.804 | 30/171/312 |
| rs7521 | A | 422/916/583 | 660/1652/938 | 0.179 | 0.95 | 1697/3754/2205 | 1734/4026/2240 | 0.357 | 0.98 | 191/380/254 | 181/463/277 | 0.641 | 119/286/150 | 479/1189/661 | 0.196 | 112/250/179 |

**Supplementary Table 2. Genotype counts, call rates and Hardy Weinberg results.** A1 = Minor Allele, Geno-A = Genotype counts in affected (LOAD), Geno-U = Genotype counts in unaffected (controls), HWE-U = Hardy Weinberg p-value in controls. Genotype counts are shown for the minor homozygote, heterozygote and major homozygote subjects. Results are shown for the Mayo cohort, and the individual JS, RS, AUT series from the Mayo cohort. Only LOAD subjects are used in the AUT series. ADGC = Alzheimer’s Disease Genetics Consortium, JS = Jacksonville Series, RS = Rochester series, AUT = Autopsy Cases.

**Supplementary Table 3.**

| **CHR** | **SNP** | **A1** | **Series** | **N_A** | **N_U** | **MAF_A** | **MAF_U** | **OR** | **L95** | **U95** | **P** |
| --- | --- | --- | --- | --- | --- | --- | --- | --- | --- | --- | --- |
| 17 | rs1467967 | G | JS | 831 | 905 | 0.333 | 0.319 | 1.10 | 0.94 | 1.28 | 0.241 |
|  |  |  | RS | 536 | 2213 | 0.355 | 0.334 | 1.11 | 0.96 | 1.28 | 0.172 |
| 17 | rs242557 | A | JS | 828 | 932 | 0.383 | 0.368 | 1.05 | 0.91 | 1.22 | 0.502 |
|  |  |  | RS | 460 | 2201 | 0.364 | 0.383 | 0.95 | 0.81 | 1.10 | 0.488 |
| 17 | rs3785883 | A | JS | 841 | 943 | 0.177 | 0.169 | 1.13 | 0.94 | 1.36 | 0.204 |
|  |  |  | RS | 578 | 2350 | 0.192 | 0.188 | 1.04 | 0.87 | 1.23 | 0.686 |
| 17 | rs2471738 | T | JS | 851 | 947 | 0.220 | 0.206 | 1.09 | 0.91 | 1.30 | 0.366 |
|  |  |  | RS | 585 | 2355 | 0.222 | 0.211 | 1.07 | 0.90 | 1.26 | 0.451 |
| **17** | **rs8070723** | **G** | **JS** | **838** | **939** | **0.213** | **0.241** | **0.81** | **0.68** | **0.96** | **0.018** |
|  |  |  | **RS** | **552** | **2287** | **0.187** | **0.214** | **0.81** | **0.68** | **0.96** | **0.019** |
| 17 | rs7521 | A | JS | 825 | 921 | 0.462 | 0.448 | 1.08 | 0.94 | 1.25 | 0.289 |
|  |  |  | RS | 555 | 2329 | 0.472 | 0.461 | 1.06 | 0.92 | 1.23 | 0.404 |

**Supplementary Table 3. *MAPT* single SNP association results with LOAD risk in the individual Mayo Clinic series. Results of multivariable logistic regression analysis.** A1 = Minor Allele, N= number of subjects with genotype calls, A = Affected (LOAD), U = Unaffected (Control), MAF = Minor Allele Frequency, OR = Odds Ratio, L95 and U95 = Lower and Upper 95% Confidence intervals, P = p-value.

**Supplementary Figure 1.**


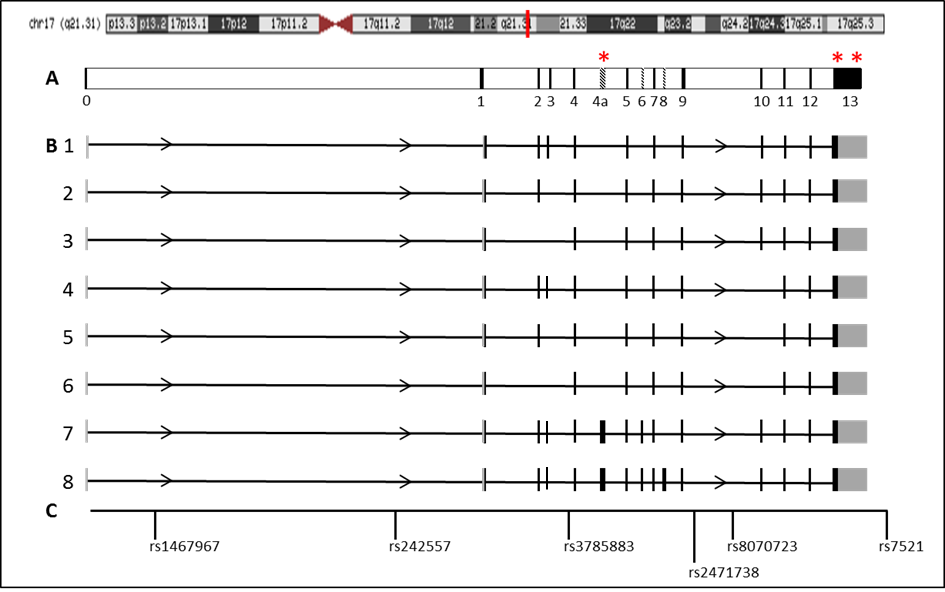


**Supplementary Figure 1. *MAPT* Refseq mRNA isoforms and SNP annotation. (A)** The position of *MAPT* on Chromosome 17 is shown with a red vertical line. The structure of the gene is shown with exons (0 -13) in solid black or striped boxes and introns in white. Red asterix (*) indicates exons to which DASL probes bind. Probes ILMN_1710903 and ILMN_2310814 target exon 13 common to all transcripts, ILMN_2298727 targets exon 4a. **(B)** Eight mRNA transcripts are shown with black boxes representing coding exons and grey boxes un-translated regions. (1) Isoform 1, NM_005910, (2) Isoform 2, NM_001123067, (3) Isoform 3, NM_016834, (4) Isoform 4, NM_001203252, (5) Isoform 5, NM_001203251, (6) Isoform 6, NM_016841, (7) Isoform 7, NM016835.4, (8), Isoform 8, NM_01123066.3. Arrows indicate the direction of transcription. **(C)** The relative positions of the six haplotype tagging SNPs genotyped in this study are shown. Figure adapted from UCSC genome browser ([www.genome.ucsc.edu](http://www.genome.ucsc.edu/), 091912)

**Supplementary References:**

1. Carrasquillo MM, Zou F, Pankratz VS, Wilcox SL, Ma L, Walker LP *et al.* Genetic variation in PCDH11X is associated with susceptibility to late-onset Alzheimer's disease. *Nat Genet* 2009; **41**(2)**:** 192-198.

2. Pittman AM, Myers AJ, Abou-Sleiman P, Fung HC, Kaleem M, Marlowe L *et al.* Linkage disequilibrium fine mapping and haplotype association analysis of the tau gene in progressive supranuclear palsy and corticobasal degeneration. *J Med Genet* 2005; **42**(11)**:** 837-846.
